# Cellulitis Caused by *Hirudo orientalis* Bites That Lead to an Allergic reaction

**DOI:** 10.1155/2022/5493057

**Published:** 2022-10-22

**Authors:** Mohsen Najjari, Rahmat Solgi, Amir Tavakoli Kareshk

**Affiliations:** ^1^Department of Parasitology and Mycology, Faculty of Medicine, Mashhad University of Medical Sciences, Mashhad, Iran; ^2^Infectious Diseases Research Center, Birjand University of Medical Sciences, Birjand, Iran; ^3^Student Research Committee, Birjand University of Medical Sciences, Birjand, Iran

## Abstract

The allergic reaction due to leech bites is frequently reported due to complications of leech therapy and also unwanted leech infestation. Regularly, the urticarial papules are common, and itching lasts less than 24 h. In the case of leech biting, dermal infection could be caused by leech gut bacterial flora such as Aeromonas spp and histamine from leech saliva. In this case report, a 30-year-old diabetic woman, who works in the field of leech breeding, was bitten by *Hirudo orientalis* during breeding. Her clinical signs were inflammation, swelling, pain, and redness in the back of her left hand. A microbiological examination revealed that the isolated leech was infected with *Aeromona hydrophila*. The risk of death due to anaphylactic shock and sepsis is high in some cases of underlying diabetes and immunocompromised individuals. The study pointed out the hazards of leech bites and proposed preventative measures such as using gloves and boots for farm workers.

## 1. Introduction

An allergic reaction due to leech bites is frequently reported due to complications of leech therapy and unwanted leech infestation [1]. Regularly, the urticarial papules are common, and itching lasts less than 24 h. Dermal infection may be caused by leech gut bacteria flora such as Aeromonas spp. and histamine in leech saliva in the case of leech biting [2]. Leeches belong to the phylum Annelida and the class Clitellata [3]. Their bodies are muscular and pigmented, with a tough cuticle, suckers at both ends, hard jaws, and a muscular pharynx. A large number of leeches live in freshwater environments such as rivers and ponds, while some species can be found in terrestrial and marine environments. Most of them are predominantly bloodsuckers from vertebrate and invertebrate animals [4]. However, severe injuries to the internal viscera by leech bites are uncommon [5, 6]. When they do occur, they can cause serious morbidity and may even have fatal consequences [7]. Leech bites can also involve various organs such as the esophagus, larynx, and pharynx [8]. Leech breeders and employees involved in outdoor water activities are more vulnerable to leech bites. Allergies and cellulitis are common and can be classified as occupational diseases [9]. In some cases, such as diabetics and those with weakened immune systems, the risk of death from anaphylactic shock and sepsis is high [10, 11]. This study identified the risks of leech bites in leech breeders, such as people who sell and handle leeches, as well as leech therapy and unwanted leech infestation.

## 2. Case Presentation

**Figure 1:**
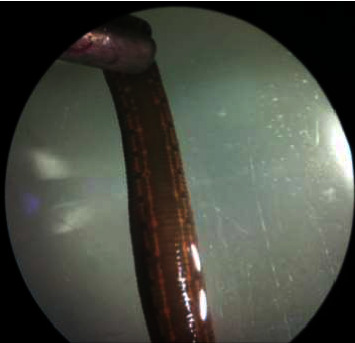
The morphological feature of the isolated leech as *Hirudo orientalis*.

In August 2018, a 30-year-old diabetic woman with complaints of inflammation, swelling, pain, redness in the back of her left hand (Figure 1), and chest tightness was referred to Mashhad's Ghaem Hospital. The patient works in the field of leech breeding and was bitten by leech during breeding. The patient's Y-shaped wound had been healing for over 5 days, and she was in extreme pain. The isolated leech was washed several times with normal saline. For morphological identification of the leech, the isolated samples were relaxed with pure ethanol. Morphological identification was carried out using a stereomicroscope (Leica EZ4, Germany) in accordance with the standard keys [12]. The morphological study was approved by a molecular study. In brief, DNA extraction was performed by using a DNA extraction kit. The partial cytochrome c oxidase subunit 1 (COI) DNA fragment of 709 bp was amplified using the previously published LCO 1490 and HCO 2198 primers [13]. In the Medical Microbiology Laboratory of Mashhad University of Medical Sciences, a microbiological examination was performed to detect the bacterial infectious agent in wound secretions.

**Figure 2:**
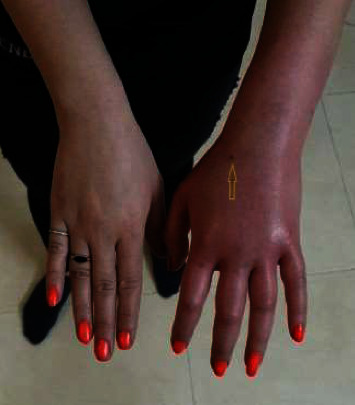
Arrows show inflammation, swelling, and redness in the metacarpal of the left hand.

The isolated leech has a large cylindrical body with a length of 80–90 mm. The anterior sucker was larger than the posterior one, and the dorsal view revealed a strip separated by circular patches. These characteristics were linked to *Hirudo orientalis* (Figure 2). A molecular study was carried out to determine the morphological finding. Sequencing results from both the forward and reverse strands were obtained. The isolated leech was successfully sequenced, and nucleotide BLAST analysis of 709 bp of the COI gene sequence revealed that it belonged to *H. orientalis* with 99 percent homology (OL759130). *Aeromona hydrophila* was identified through microbiological testing. Ciprofloxacin 500 mg daily (for 2 weeks) and dexamethasone 4 mg/ml injections were prescribed in accordance with the standard protocol.

## 3. Discussion

Leech infestation is reported regularly due to complications of unwanted leech biting or leech therapy. Some scientists suggested that the medical leeches used in medical fields must be evaluated for pathogen contamination before using. Cellulitis has been the most common clinical manifestation, with an incidence ranging from 4.1 to 36.2 percent [14]. The use of medical leeches to treat human diseases has a long history, especially in Iran [2]. The previous study detected the main medical leeches (*Hirudo orientalis, Hirudo medicinalis, and Hirudo verbana*) in the field of medicine in Iran [15]. According to our knowledge, leech saliva contains bioactive substances such as hirudin, calin, hyaluronidase, histamine-like substances, and a variety of other proteins [16]. It has been effectively utilized in plastic and reconstructive medical procedures, cardiovascular intricacies, varicose veins, hemorrhoids and different joint infirmities, gastrointestinal problems, dermatology, and gynecological anomalies. Recently, hirudo therapy has found new applications in the treatment of some malignancies and extreme touchiness conditions similar to asthma and diabetes [11–14, 16, 17]. Despite leech's beneficial properties in the treatment of some human diseases in some cases, accidental leech bites or medical leech therapy can also cause a skin infection. The symptoms are malaise, painless bleeding, bruising, itching, burning, irritation, and redness [18]. It has been established that all leeches carry symbiotic bacteria in their intestines, such as A. hydrophila [19]. The accidental excretion of the intestinal bacteria of the leech during the process of attaching may lead to the onset of an allergic reaction [20]. *A. hydrophila* can cause severe local skin infections in a variety of patterns. Folliculitis (pustules), abscesses, impetigo-like rash, tarsal gangrene, cellulitis, redness, and swelling affecting the deeper skin, and necrotic fasciitis are some of the clinical signs. [21]. Aeromonas sepsis is potentially dangerous [22]. The cutaneous infections caused by Aeromonas are more likely to cause serious complications in immunocompromised patients [23, 24]. Some strains of *A. hydrophila* can produce aerolysin toxins, which lead to tissue damage. *A. hydrophilic* infections occur when bacteria enter damaged areas of the skin, due to razors, abrasions, surgical wounds, insect bites, and leech bites [25]. In this study, the bacterial infection has been isolated from the secretions of the wound and detected as an *A. hydrophilic* infection by gram stain. *A. hydrophilic* infection was approved by the culture and molecular techniques. Some Aeromonas strains are multidrug-resistant, so an antibiotic susceptibility test is required before prescribing antibiotics if necessary [15, 26]. Leech breeders and others involved in aquatic activities are more vulnerable to unwanted leech bites. Allergic reactions and cellulitis are common and may be considered occupational diseases [27]. As previously reported in the literature, the risk of death due to anaphylactic shock and sepsis is high in some cases, such as our underlying diabetic case and people with immune deficiency disorders [11]. The study highlighted the dangers of leech bites and suggested preventative measures such as wearing gloves and boots in leech breeding facilities to reduce the risk of such allergic reactions. Finally, in emergency situations, leech detachment is required. Simple methods such as perfuming a strong salt solution, lighting a match, consuming alcohol, and others suggested in the articles can be used [5].

## Data Availability

No data were used to support the findings of this study.
